# Unrecognized abdominal pregnancy with six months’ evolution revealed by acute intestinal obstruction in women with PCOS

**DOI:** 10.5935/1518-0557.20230057

**Published:** 2024

**Authors:** Sana Ghades, Abderahmen Daadoucha, Hamed Jemel, Nour Rouis, Mohamed Ridha Fatnassi

**Affiliations:** 1 Department of Gynecology and Obstetrics, University Hospital Ibn El Jazzar, 3100, Kairouan, Tunisia; 2 Department of Radiology, University Hospital Ibn El Jazzar, 3100, Kairouan, Tunisia

**Keywords:** Pregnancy, Abdominal, Douglas’, Pouch, Hemoperitoneum, Ultrasound, Imaging

## Abstract

Abdominal pregnancy is a rare form of ectopic pregnancy where implantation and development of the egg take place in the peritoneal cavity outside the tubo-uterine mucosa, in contact with intestinal loops. Diagnosis is most often difficult. We report the case of a 32-year-old woman (gravida 1, para 1), with a history of PCOS, diagnosed with abdominal pregnancy at 20 weeks of amenorrhea complicated by acute intestinal obstruction. Diagnosis was confirmed by abdomino-pelvic scan. Surgery was performed with the patient under general anesthesia. She presented a macerated fetus with an infiltration of the placenta causing a perforation of the sigmoid colon and uterus. Hartmann’s procedure was performed and the perforation of the uterus was sutured. Abdominal pregnancy remains a rare variety of ectopic pregnancy. Preoperative diagnosis is difficult due to the presence of a variety of non-specific symptoms. This type of ectopic pregnancy remains challenging for gynecologists and radiologists.

## INTRODUCTION

Abdominal pregnancy (AP) is generally defined as an ectopic pregnancy where implantation and development of the egg take place in the peritoneal cavity outside the tubo-uterine mucosa, in contact with intestinal loops. It may develop for five months or longer (Aliyu & Ashimi, 2013). The frequency of abdominal pregnancy is estimated at 1-2% of all ectopic pregnancies (Oudghiri *et al*., 2013). Diagnosis is most often difficult given the variety of clinical signs, which vary according to age and location of the abdominal pregnancy. Progression to term without incident is rare, but possible.

We report a case of abdominal pregnancy found after a patient sought care for acute intestinal obstruction.

## CASE REPORT

A 32-year-old woman (gravida 1, para 1) with one vaginal delivery arrived at the emergency department complaining of acute abdominal pain and cessation of matter and gas for three days. The patient (weight: 95kg, body mass index (BMI): 32.87kg/m^2^) had secondary infertility due to polycystic ovary syndrome (PCOS), for which she has been followed by a private doctor since 2021.

She had been married for five years and gave birth to a baby boy in 2018 from a spontaneous pregnancy. She experienced irregular cycles and spaniomenorrhea. She had a menstrual cycle of 45 to 120 days and a period duration of 4 to 5 days. She had been treated with drugs and could only adjust her menstrual cycle with medication. She frequently used non-hormonal contraception with her partner (such as condoms and spermicide). Serum hormone levels including LH and FSH were in favor of PCOS. Anti-Müllerian hormone (AMH) levels were normal. A transvaginal ultrasound scan showed multiple antral follicles of predominantly 4-10 mm in size in both ovaries. Biological tests, combined with clinical diagnosis and the characteristics of the patient’s infertility, suggested PCOS.

She received two cycles of ovarian stimulation with clomiphene citrate without achieving pregnancy. Her last cycle was eight months before she sought care for abdominal pain. She stopped treatment because of conflicts with her husband. She reported spotting with unusual cycles.

The patient complained of abdominal pain, nausea, vomiting and constipation for the past five months. She consulted her doctor and her symptoms were reported as part of a functional colopathy. She did not undergo ultrasound examination. However, she ignored her pregnancy because she had a history of irregular menstrual periods. She did not use contraceptive methods because she had been separated from her husband for four months.

At admission, she was in good general condition with a temperature was 37.5°C and normal blood pressure and pulse rate. Physical examination found a distended painful acute abdomen without vaginal bleeding or cervical modification. Digital rectal examination revealed an empty rectal ampulla. Laboratory parameters showed a hemoglobin concentration of 10.0 g/dl, hematocrit of 32.1% and CRP of 90 mg/l.

**Figure 1:**
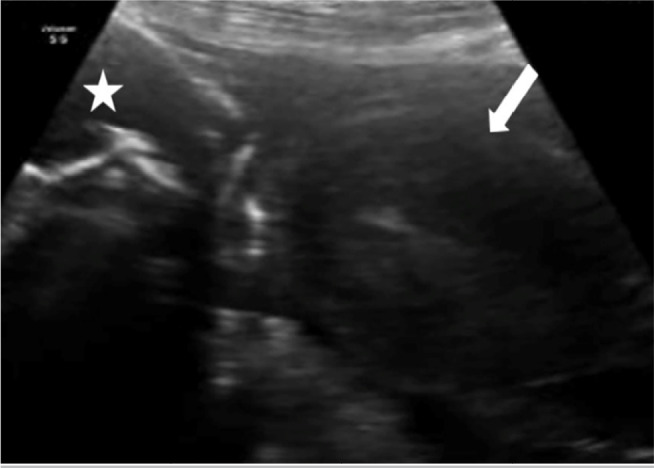
Trans-abdominal ultrasound scan showing a fetus (star) separate from the uterus (arrow) and close to the mother’s anterior abdominal wall.

**Figure 2:**
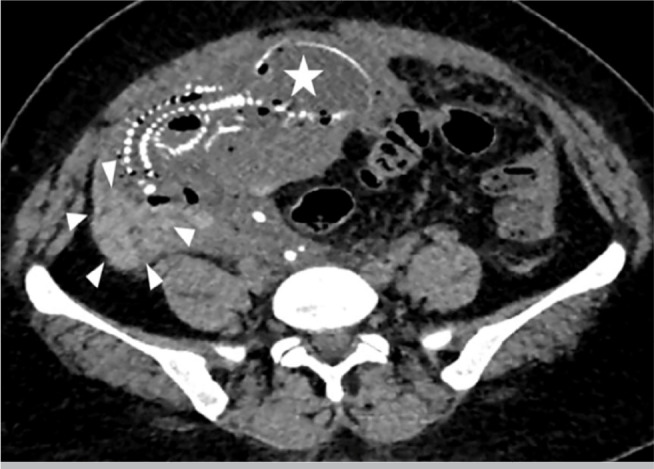
Abdomino-pelvic scan showing the fetus (star) and the placenta (arrow) in the abdominal cavity outside the uterus.

The emergency sonographic evaluation founded an empty uterus and a fetus on the right rear side of the abdominal cavity, not surrounded by uterine muscle tissue (Figure 1). The fetal heartbeat was invisible and the bones of the skulls were overlapped. There was an obvious collection of free fluid in the pelvis. Magnetic resonance imaging (MRI) was not available. An abdomino-pelvic scan showed an abundant hemoperitoneum and an abdominal pregnancy after 20 weeks of amenorrhea without cardiac activity (Figure 2).

**Figure 3:**
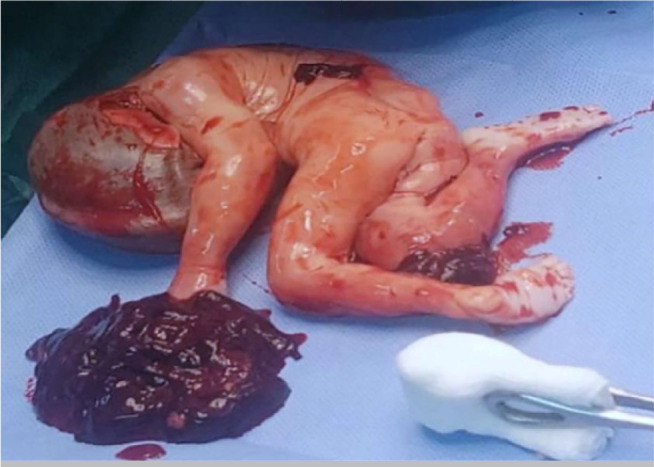
Picture of the fetus and placenta after extraction.

**Figure 4:**
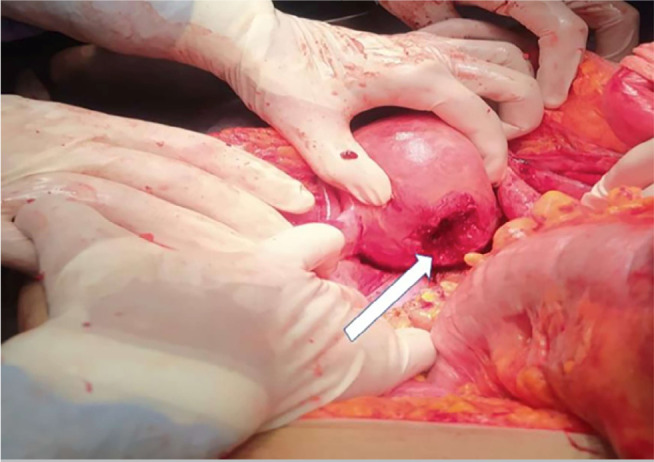
Intraoperative discovery of sigmoid perforation with uterine rupture (arrow) after extraction of the placenta.

The patient’s clinical condition became worse and an emergency laparotomy was performed. During the operation, we found a hemoperitoneum of 1000 ml, a soft and friable encapsulated retro-uterine mass of about 20cm strongly adhered to the mesosigmoid, the sigmoid, and the posterior wall of the uterus. The mass sheathed the sigmoid colon and the pouch of Douglas. A one-piece excision of the mass was performed. The break-in of which showed a placenta attached to the mesosigmoid connected to the fetus and clots of about 500ml (Figure 3). We also noted a perforation of the sigmoid colon and the posterior wall of the uterus (Figure 4).

Hartmann’s procedure was performed with surgical resection of the rectosigmoid colon, closure of the anorectal stump, and formation of an end colostomy. Suture of uterine perforation was made after toilet and uterine revision. Postoperative management was uneventful and the restoration of digestive continuity will be scheduled after three months. The bhCG tests performed on postoperative day 3 and one month later were negative. The patient has no discomfort and is still under close follow-up.

## DISCUSSION

Abdominal pregnancy (AP) occurs in the peritoneal cavity when the egg is fertilized and begins developing directly in contact with the bowel. It occurs in two forms:

- a secondary form, the most frequent, linked to tuboabdominal abortion or ruptured tubal ectopic pregnancy (Le Lorier *et al*., 1969)
- and a primary form, of rare occurrence, since it must meet the Studdiford criteria (Studdiford, 1942) as follows: normal fallopian tubes and ovaries, absence of uteroperitoneal fistula and exclusive contact of the egg with the peritoneal surface.

In our patient, the presence of pregnancy in the pouch of Douglas led to description of the case as a secondary form. Although more common than the primary form, it remains a rare variety of ectopic pregnancy, with perinatal mortality ranging between 40% and 95% (Guèye *et al*., 2012). Risk factors for ectopic pregnancy include sterility, intrauterine device, history of uterine trauma, termination of pregnancy by aspiration, uterine scar, genital infections (Oudghiri *et al*., 2013). Our patient had risk factors for ectopic pregnancy such as secondary sterility and ovarian stimulation.

The frequency of AP is high in developing countries (1/2000 deliveries) (Guèye *et al*., 2012; Bohoussou *et al*., 2013), unlike in developed countries, where this type of pregnancy is rare (1/10 000-1/15 000 deliveries) and generally discovered at an early stage (Takeda *et al*., 2012). The difference is explained by the risk factors associated with socio-economic factors, such as availability of medically assisted procreation and use of intrauterine devices in developed countries versus the high incidence of genital infection and insufficient monitoring of pregnancy in developing nations (Guèye *et al*., 2012; Bohoussou *et al*., 2013; Takeda *et al*., 2012).

The preoperative diagnosis of AP is difficult due to the occurrence of non-specific clinical signs and symptoms linked to diagnosis (Oudghiri *et al*., 2013). However, for some authors, continuous abdominal pain associated with amenorrhea are the main symptoms. AP may at times manifest with hemoperitoneum, peritonitis and intestinal obstruction (Kangulu *et al*., 2013), as in the case described herein.

Ultrasound examination plays a key role in the identification of clinical signs of AP. Indeed, in more than 50% of cases it enables the establishment of preoperative diagnosis when an empty uterus and an absence of uterine wall around the fetus, which is in contact with the intestinal echoes, are seen (Diagnosis and Management of Ectopic Pregnancy: Green-top Guideline No. 21, 2016; Gerli *et al*., 2004). The abdomino-pelvic scan is contraindicated during pregnancy, but our patient presented a non-viable fetus and MRI was not available in the emergency department of our service. The abdomino-pelvic scan was ordered as part of an OIA feature and allowed us to confirm the diagnosis. However, MRI offers a decisive contribution in establishing diagnosis (Oudghiri *et al*., 2013).

The treatment of AP is always surgical. However, surgery can be postponed to around the period of fetal viability, provided that maternal and fetal monitoring is reinforced (Nzaumvila *et al*., 2018). In our case, the absence of viability of the fetus and the feature of OIA required the realization of a laparotomy. Laparoscopic surgery is, according to the literature, feasible in cases of pregnancy of less than 12 weeks and implantation of this pregnancy should be compatible with laparoscopic surgery (Rabarikoto *et al*., 2018). Complete delivery must be achieved after inventorying the relationship between the placenta and the pelvic-abdominal organs. The placenta can be left in situ after clamping the cord flush with the placental surface. Its resorption can be monitored by Doppler ultrasound and/or with plasma beta-HCG tests (Hamouda *et al*., 2013). In our patient, the implantation on the mesosigmoid with perforation of the sigmoid imposed an excision and a colostomy. Diagnosis is confirmed by histological examination (Collège National des Gynécologues et Obstétriciens Français, 2005), as was the case in our patient.

## CONCLUSION

Abdominal pregnancy (AP) remains a rare variety of ectopic pregnancy. The symptoms are non-specific and variable. Acute intestinal obstruction is one of the rare varieties. This case explores the main issues and challenges faced by gynecologists and radiologists. One of the first challenges was to make the diagnosis of AP in a patient with abdominal pain associated with amenorrhea previously diagnosed with PCOS. The role of the radiologist is crucial in the choice of the most appropriate management. Conservative therapeutic approaches have been proposed in both complicated and uncomplicated second-trimester abdominal pregnancies.

## References

[r1] Aliyu LD, Ashimi AO. (2013). A multicentre study of advanced abdominal pregnancy: a review of
six cases in low resource settings. Eur J Obstet Gynecol Reprod Biol.

[r2] Bohoussou E, Guié P, Saki C, Okon G, Anongba S, Touré-Coulibaly K. (2013). Diagnosis and management of advanced abdominal pregnancy
(Abidjan, Côte d’Ivoire). Rev Int Sci Med Abidjan.

[r3] (2005). Collège National des Gynécologues et
Obstétriciens Français. J Gynecol Obstet Biol Reprod (Paris).

[r4] (2016). Diagnosis and Management of Ectopic Pregnancy: Green-top
Guideline No. 21. BJOG.

[r5] Gerli S, Rossetti D, Baiocchi G, Clerici G, Unfer V, Di Renzo GC. (2004). Early ultrasonographic diagnosis and laparoscopic treatment of
abdominal pregnancy. Eur J Obstet Gynecol Reprod Biol.

[r6] Guèye M, Cissé ML, Guèye SMK, Guèye M, Diaw H, Moreau JC. (2012). Difficultés du diagnostic et de prise en charge de la
grossesse abdominale: à propos de deux cas diagnostiqués
à terme au Centre Hospitalier Régional de Diourbel du
Sénégal. Clin Mother Child Health.

[r7] Hamouda ES, Littooij AS, Thia EW, Ong CL. (2013). Ruptured interstitial ectopic pregnancy at 18 weeks gestation
diagnosed by MRI: a case report. J Radiol Case Rep.

[r8] Kangulu IB, Umba EK, Cibuabua DK, Ilunga CM, Ndolo AU, Nzaji MK, Kayamba PK. (2013). A propos d’un cas de grossesse abdominale très
prolongée [About a case of very prolonged abdominal
pregnancy]. Pan Afr Med J.

[r9] Le Lorier G, Schebat C, Wencel S. (1969). La grossesse abdominale au voisinage du terme avec enfant vivant.
Problèmes diagnostiques et thérapeutiques: à propos
d’un cas [Abdominal pregnancy near term with a living infant. Diagnostic and
therapeutic problems: a case]. Bull Fed Soc Gynecol Obstet Lang Fr.

[r10] Nzaumvila DK, Govender I, Ogunbanjo GA. (2018). An audit of the management of ectopic pregnancies in a district
hospital, Gauteng, South Africa. Afr J Prim Health Care Fam Med.

[r11] Oudghiri N, Doumiri M, Behat M, Tachinante R, Tazi AS. (2013). Grossesse extra-utérine abdominale à
terme. Maroc Med.

[r12] Rabarikoto HF, Rakotomboahangy TM, Razafindrabia TR, Razafindratasy E, Randriambololona DMA. (2018). Grossesse abdominale: les difficultés diagnostiques
à travers un cas. Rev Anesth Reanim Med Urg Toxicol.

[r13] Studdiford WE. (1942). Primary peritoneal pregnancy. Am J Obstet Gynecol.

[r14] Takeda A, Imoto S, Mori M, Yamada J, Nakamura H. (2012). Early abdominal pregnancy complicated by parasitic dermoid cyst:
diagnosis by diffusion-weighted magnetic resonance imaging and management by
laparoendoscopic single-site surgery. J Minim Invasive Gynecol.

